# Is the Oral Microbiome Important in HIV-Associated Inflammation?

**DOI:** 10.1128/mSphere.00034-20

**Published:** 2020-02-05

**Authors:** Jennifer A. Fulcher

**Affiliations:** a Division of Infectious Diseases, Department of Medicine, David Geffen School of Medicine at UCLA, Los Angeles, California, USA; b VA Greater Los Angeles Healthcare System, Los Angeles, California, USA

**Keywords:** human immunodeficiency virus, inflammation, oral microbiology

## Abstract

Alterations in the gut microbiome during HIV infection have been implicated in chronic inflammation, but the role of the oral microbiome in this process is less clear. The article by M. K. Annavajhala, S. D. Khan, S. B. Sullivan, J. Shah, et al. (mSphere 5:e00798-19, 2020, https://doi.org/10.1128/mSphere.00798-19) investigated the relationship between oral and gut microbiome diversity and immune activation in patients with HIV on antiretroviral therapy. In this study, oral microbiome diversity was inversely associated with inflammatory markers such as soluble CD14 (sCD14), but surprisingly similar associations were not seen with gut microbiome diversity. Oral microbiome diversity was also associated with periodontitis in these patients. This study highlights the importance of continuing multisite examinations in studying the gastrointestinal tract microbiome and also stimulates important directions for future research defining the role of the oral-gut axis in HIV-associated inflammation.

## Abstract (precis)

Alterations in the gut microbiome during HIV infection have been implicated in chronic inflammation, but the role of the oral microbiome in this process is less clear. The article by M. K. Annavajhala, S. D. Khan, S. B. Sullivan, J.

## COMMENTARY

The gastrointestinal tract contains trillions of commensal microbes that are integral to immune development and function. The microbial composition, or microbiome, is shaped by the local microenvironment and varies greatly by anatomic site from the oral cavity to the large intestine. Most research has focused on the intestinal microbiome, but other sites such as the oral cavity have increasingly recognized roles in health and disease. We are now learning that the microbiome in the gastrointestinal tract is not as compartmentalized as once thought. Recently, Schmidt et al. showed that there is ongoing direct migration of bacteria from the mouth to the gut even in healthy individuals (1). What does this mean? In most individuals, there is no apparent clinical consequence of oral-to-gut bacterial migration, and this may be a necessary mechanism to shape the gut microbiome. However, some oral bacteria appear to cause gut inflammation (2–4), and multiple clinical studies have associated the presence of “oral” bacteria in the gut with inflammatory diseases (5–7), including HIV (8, 9).

The gut microbiome has been implicated in driving systemic inflammation in chronic HIV-1 infection. The loss of the critical barrier regulating Th17 cells during acute HIV-1 infection increases microbial translocation and inflammation (10–12), and indicators of microbial translocation have been repeatedly associated with systemic inflammation in clinical studies (13–15). Gut bacteria may directly contribute to HIV-related inflammation via translocation (13) or direct interaction with mucosal immune cells (16). How the oral microbiome contributes to this cascade has received relatively little attention.

Annavajhala et al. (17) address this question by comparing oral and gut microbiome diversity among persons living with HIV on suppressive antiretroviral therapy. The authors examined the oral and gut microbiomes in 52 women and men with HIV, including longitudinal sampling in a portion of the cohort. Low CD4 nadir was associated with lower gut bacterial diversity, consistent with other studies (18), but this was not seen in the oral microbiome. When examining systemic markers of inflammation, soluble CD14 (sCD14) and interleukin 6 (IL-6) were inversely associated with saliva bacterial diversity, yet no associations were found with gut bacterial diversity. This is in contrast to many other studies which have linked the gut microbiome to systemic inflammation (13–15), though none of these prior studies simultaneously examined the oral compartment. Whether this suggests a more important role of the oral microbiome in systemic inflammation or is simply a product of differences in study design, populations, and sample size is not known. It is also possible that the oral microbiome plays an important role only in certain stages of disease, and these may not coincide with the gut microbiome.

Though compelling, these findings should be considered in the context of a few important limitations. Most notably, this study is limited to people living with HIV, so it is not known how these findings compare to those without HIV. In other settings, the oral microbiome has been associated with periodontitis and systemic inflammation (19); how might HIV augment this relationship? Another important consideration is the contribution of periodontitis to these findings. In this study, saliva bacterial diversity was also associated with moderate to severe periodontitis, and specific taxa including Prevotella melaninogenica and Rothia mucilaginosa were associated with periodontitis. Interestingly, these same species were associated with HIV disease in a separate study of HIV-infected and uninfected women (20). Finally, though the study by Annavajhala et al. (17) is among the larger studies published so far investigating the role of the oral microbiome in HIV-associated inflammation, it is still limited in size. This highlights the critical need for larger, longitudinal studies with multiple site sampling for comparative analyses of microbial composition and evolution in the context of HIV and inflammation.

Importantly, the study by Annavajhala et al. (17) is one of the few published studies that has included women. No differences in bacterial diversity were seen by sex at either site in this study, but the small sample size and uneven distribution of men and women may have limited the ability to detect differences. Moving forward, a focused effort on better understanding of the role of sex on both the oral and gut microbiome in HIV will be critical.

This study adds further evidence that the effects of the microbiome on mucosal and systemic immunity are not limited to the intestinal tract. The authors challenge the notion that alterations in the gut microbiome are most influential in HIV-associated inflammation and provide data suggesting that oral microbiome diversity may play an equal or even greater role in systemic inflammation. Given the evidence that the oral and gut microbiomes interact, it is unlikely that either site functions in isolation. Defining the dynamics between different microbiome compartments, and how these populations interact, will better our understanding of how commensal microbiota influence disease. What is the timing of oral microbiome changes related to gut dysbiosis? What characteristics of the oral bacterial species lead to inflammation when migrated to a distal site? It is likely that bacteria evolve and adapt to their specific niche, and when placed in another environment cause disruption of the homeostatic ecosystem which has existed. Understanding what is different about these translocated species may give insight into the immune environment perturbations that promote dysbiosis, and potentially provide therapeutic targets to dampen this process.
